# Establishment of a CRISPR-Based Lentiviral Activation Library for Transcription Factor Screening in Porcine Cells

**DOI:** 10.3390/ani15010019

**Published:** 2024-12-25

**Authors:** Yingjuan Liang, Xiaoxia Yao, Jingxin Han, Jinpeng Wang, Xiao Zhang, Donglin Zhao, Chaoqian Jiang, Lishuang Geng, Shihao Lv, Zhonghua Liu, Yanshuang Mu

**Affiliations:** 1 Key Laboratory of Animal Cellular and Genetic Engineering of Heilongjiang Province, Northeast Agricultural University, Harbin 150030, China; yingjuanl@163.com (Y.L.); yaoxiaoxia202209@163.com (X.Y.); hanjingxin2022@163.com (J.H.); zhangxiao0529@yeah.net (X.Z.); jiangchaoqianneau@163.com (C.J.); genglishuangtracer@163.com (L.G.); s220901084@neau.edu.cn (S.L.); 2 College of Life Science, Northeast Agricultural University, Harbin 150030, China; zhaodonglin0330@163.com; 3 Key Laboratory of Public Nutrition and Health, National Health Commission of the Peoples’ Republic of China, National Institute for Nutrition and Health, Chinese Center for Disease Control and Prevention, No. 155 Changbai Road, Changping District, Beijing 102206, China; lpl6661010@163.com

**Keywords:** porcine, CRISPR activation library, transcription factor, OCT4

## Abstract

Transcription factors play important roles in the growth and development of various tissues in pigs, such as muscle, fat, and bone. A transcription-factor-scale activation library based on the clustered, regularly interspaced, short palindromic repeat (CRISPR)/CRISPR-associated endonuclease Cas9 (Cas9) system could facilitate the discovery and functional characterization of the transcription genes involved in a specific gene network. Here, we have designed and constructed a CRISPR activation (CRISPRa) sgRNA library, containing 5056 sgRNAs targeting the promoter region of 1264 transcription factors in pigs. The sgRNA library, including sgRNA with MS2 loops, is a single-vector system and is packaged with lentivirus for cell screening. Porcine PK15 cells expressing the porcine OCT4 promoter driving EGFP, dCas9 fused with VP64, and MS2-binding protein-p65-HSF1 were constructed, and then, the sgRNA activation library was used to screen the transcription factors regulating OCT4 expression. After the lentiviral transduction and deep sequencing of the CRISPR sgRNAs library, the highest ranking candidate genes were identified, including 31 transcription factors activating OCT4 gene expression and 5 transcription factors inhibiting OCT4 gene expression. The function and gene regulation of the candidate genes were further confirmed by the CRISPR activation system in PK15 cells. The CRISPR activation library targeting pig transcription factors provides a promising platform for the systematic discovery and study of genes that determine cell fate.

## Simple Summary

Pigs are important farm animals, and studying pig transcription factors can help us to understand the molecular mechanisms related to the key economic traits of pigs. The establishment of a whole pig transcription factor sgRNA library based on CRISPR activation technology will facilitate the discovery of the key transcription factors involved in specific functions. In this study, we constructed a whole pig transcription factor sgRNA library and screened it for transcription factors that activate and inhibit the OCT4 gene. Our CRISPR activation library targeting whole pig transcription factors provides an unbiased discovery approach for the systematic identification of transcription factors that drive cell fate acquisition.

## 1. Introduction

Pigs are high-quality animal protein sources for people in many countries, and the growth and development rates of muscle traits, fat traits, and reproductive traits are the major selection indicators used in pig genetic breeding [1]. Each trait in pigs has a major transcription regulatory factor, such as myogenic differentiation 1 (MyoD1), the main transcription factor for muscle development; PPARγ, the main transcription factor for fat development; and OCT4, the main transcription factor for embryonic development [2,3,4]. These transcription factors are important candidate genes for genetics and breeding [5]. By better understanding the regulatory networks of these transcription factors, more precise breeding strategies can be developed.

CRISPR activation (CRISPRa) screens offer a powerful unbiased and high-throughput approach to identifying the key transcription factors and pathways that underlie biological processes [6,7]. The CRISPR activation system activates endogenous gene expression by fusing dCas9 with a transcription activator and targeting the endogenous gene promoter region mediated by gRNA [8]. With the development of CRISPR activation technology, the dCas9-SunTag system [9], dCas9-VPR system [10], and dCas9-SAM system [6] were developed and were found to be more suitable for genome-wide activation screening. Genome-wide CRISPRa libraries have been successfully used for gain-of-function screening in several cell types, including human neurons, human melanoma cell lines, and mouse embryonic stem cells (ESCs) [11,12,13,14,15,16]. When combined with single-cell transcriptome sequencing, CRISPRa screening identified the key factors that induce zygotic genome activation transcription in ESCs [17]. The advantages provided by CRISPR-based gRNA libraries over open reading frame (ORF) libraries are becoming increasingly apparent: sgRNA is smaller than cDNA, so sgRNA libraries are easier to design and construct and have lower costs than cDNA libraries. SgRNAs are more easily transmitted to target cells than cDNA and can upregulate the expression of gene isoforms and the non-coding genome [18,19]. CRISPRa screens using gRNA libraries offer an efficient, scalable, and cost-effective method to investigate gene function and regulatory mechanisms, providing insights into complex biological processes and facilitating advances in functional genomics [12,19].

OCT4 (Octamer-binding transcription factor 4) is a critical regulator of pluripotency and cell differentiation during early embryonic development. It maintains the undifferentiated state of embryonic stem cells (ESCs) and plays a core role in the regulation of pluripotency [20]. The expression level of OCT4 is a key determinant in the fate of pluripotent cells: low expression supports self-renewal, moderate expression is essential for maintaining pluripotency, and high expression triggers differentiation [21]. In embryonic development, OCT4 regulates the differentiation of the ectodermal and endodermal lineages. The absence of OCT4 expression in early embryos leads to the differentiation of ESCs into trophoblast cells and the loss of pluripotency in the inner cell mass (ICM) [22,23]. A mutation in OCT4 impairs blastocyst growth and prevents ESCs from maintaining an ectoderm-like phenotype [24]. The precise control of OCT4 expression is critical for ensuring proper development and differentiation [25]. Understanding the transcriptional mechanisms that regulate OCT4 expression is vital for advancing pluripotency regulation in pig embryonic stem cells and improving in vitro embryo culture systems for higher-quality pig breeding [20].

Here, based on the CRISPRa library technology, a transcription factor sgRNA library was constructed in pigs. The sgRNA library was used to screen activation and inhibition TFs for OCT4. Using deep sequencing analysis, we identified the transcription factors regulating the expression of OCT4 in pigs and have provided a favorable reference for studying the molecular mechanism of porcine OCT4 transcription regulation. The full pig transcription factor library established in this study can be suitable for different types of cells, providing an important tool for screening the main transcription factors in different tissue cells of pigs.

## 2. Materials and Methods

### 2.1. Design of the Pig Transcription Factor sgRNA Library

A total of 1264 transcription factors (TFs) were selected based on human transcription factor data from the TRANSFAC database [26,27], combined with pig genome information from the NCBI database and g:Profiler database [28]. The transcription start site (TSS) for each gene was determined using the Gencode [29] and refFlat databases. Different isoforms of TF genes were considered as distinct TFs.

The sgRNA was designed in the range of 50 bp downstream and 3000 bp upstream of the TSS. The TSSs of transcription factors were identified in the gene database of the NCBI sub-library. Based on the TSS location information, the promoter.java script was used to extract a 3050 bp sequence of the pig genome (Taxonomy ID: 9825; reference genome: Sscrofa11.1), from 3000 bp upstream to 50 bp downstream of the TSS. SgRNAs were designed using crispor sgRNA from Zhang Feng Laboratory (http://crispor.tefor.net/, accessed on 13 December 2024). The 23 bp sequences with an NGG PAM of sgRNA were searched in the promoter region of each transcription factor in a pig (Taxonomy ID: 9825; reference genome: Sscrofa11.1). To effectively activate the expression of target genes, four sgRNAs were designed in the promoter region of each transcription factor. The library contained a total of 5056 sgRNAs, targeting 1264 transcription factors. The pig transcription factors and sgRNA sequences are shown in Supplementary Table S1.

### 2.2. Construction of sgRNA Lentivirus Library

Oligos encoding the sgRNA library with ~5046 specific sgRNA sequences targeting 1264 transcription factors were synthesized via a programmable microarray using the Synthesizer and cloned as a pool into the pLenti_sgRNA (MS2) vector (GenScript, Nanjing, China). Library quality was assessed by NGS with a coverage of 100% (GenScript, Nanjing, China). The packaging of the sgRNA library was completed by the Nanjing Genscript company.

### 2.3. Cell Culture

PK15 porcine kidney cells were cultured in DMEM (Gibco, Grand Island, NY, USA) containing 15% fetal bovine serum (Gibco, Grand Island, NY, USA) at 37 °C in 5% CO_2_ in a humidified incubator.

### 2.4. Establishment of a pOCT4-EGFP Knock-In ROSA26 Site Cell Line

An OPGN2 plasmid, in which enhanced green fluorescent protein (EGFP) was driven by the porcine OCT4 promoter, was kindly provided by Liangxue Lai [30]. Two homology arms were added to the OPGN2 plasmid by homology recombination (GenScript, Nanjing, China). The OPGN2 with two homology arms was enzymatically linearized as the donor DNA template.

PK15 cells were electroporated with the ribonucleoprotein (RNP) complex and a donor template. The sequence of the sgRNA targeting the porcine ROSA26 locus was 5′-GTG AGA GTT ATC TGA CCG TA-3′ [31]. Briefly, for 1 × 10^6^ cells, 10 μg Cas9 protein and 100 pmol sgRNA were pre-mixed for 10 min at room temperature, before adding 5 μg donor template and opti MEM (Gibco, Grand Island, NY, USA) up to a volume of 20 μL. Using a CUY21EDIT II electroporator (BEX Co., Ltd., Tokyo, Japan), the electroporation program was enacted as follows: voltage 200 V, duration 1 ms, and 5 pulses. After electroporation and the reseeding of transfected cells at a low cell density, PK15 cells were cultured for about 2 weeks to form single-cell colonies. The selected cell colonies were picked up for propagation in an independent well, and the knock-in pOCT4-EGFP in the pig ROSA26 site was PCR-amplified using primers, as shown in Supplementary Table S2-1, before being Sanger-sequenced to confirm the target DNA. The cell colonies with pOCT4-EGFP in the ROSA26 site in targeted genomic regions were selected for further functional assays.

### 2.5. Construction of dCas9-SAM System Integration pOCT4-EGFP Knock-In Cell Lines

The lentivirus packaging plasmid of the dCas9-SAM system was provided by the laboratory of cell and genetic engineering, Northeast Agricultural University. The dCas9- SAM system was packaged as a lentivirus and transduced into the pOCT4-EGFP knock-in cell line by adding lentivirus directly to the culture medium for 24 h. After 48 h, puromycin (Absin, Shanghai, China) was added for cell screening.

The genomic DNA of cells was extracted according to the operational instructions of the genomic DNA Extraction Kit (Takara, Dalian, China). Primers were designed to amplify the target fragment, as shown in Supplementary Table S2-1. The enzyme used for PCR amplification was the KOD high-fidelity enzyme (Toyobo, Dalian, China). The PCR products were sequenced to identify the target DNA.

### 2.6. sgRNA Lentivirus Library Screen

The lentiviral sgRNA library was transduced into pOCT4-EGFP cells expressing the dCas9-SAM system at an MOI of 0.3 to ensure that most cells received only one sgRNA. The cells infected with the virus were cultured in a carbon dioxide incubator at 37 °C and 5% CO_2_ for 24 h. After transduction for 2 days, 800 µg/mL bleomycin (Sigma, Shanghai, China) was added to screen and amplify the positive transduction cells for 7 days.

Flow cytometry (BD, USA) was used to analyze and screen the 15% of cells with the brightest fluorescence intensity and the 15% of cells with the lowest fluorescence intensity. Before sorting, 2 × 10^6^ cells were taken to represent a large number of unselected populations. In the process of screening, cells that were not infected with the transcription factor library were selected as the control for gate construction. We set two doors with 15% maximum and 15% minimum fluorescence intensities. The EGFP was directed using an FITC channel, and the positive cells were classified according to the position of the door.

The DNA of the cell population, sorted using flow cytometry, was extracted using a genomic DNA extraction kit (Takara, Dalian, China). Then, the sgRNA fragments were amplified by PCR using the following primers: screen-sgRNA-F, 5′-GGA CTA TCA TAT GCT TAC CGT AAC TTG-3′; screen-sgRNA-R, 5′-CCA AGT TGA TAA CGG ACT AGC CTT AT-3′. The PCR amplification products were deep-sequenced (Annoroad Gene Technology Company, Hangzhou, China).

### 2.7. Analysis of CRISPRa Screen sgRNA Data

The raw data were first processed to remove sequencing adapters using the MAGeCK algorithm (v0.5.9), which is a model-based tool designed for genome-wide CRISPR/Cas9 knockout analysis. Then, the data were mapped to sgRNA libraries to calculate normalized sgRNA counts and fold changes between selected and unselected cell pools [32]. Candidate sgRNAs and genes were screened out by filtering the sgRNAs present in all selected cell pools. The count and fold change of sgRNAs were compared with those of the unselected control group.

Individual gRNA enrichment was determined using the DESeq2 [33] package to compare gRNA abundance between high and unsorted or low and unsorted. If the gRNA of TF is significantly enriched in cells with high EGFP relative to unsorted cells (fdr < 0.01), the TF is selected as a hit.

### 2.8. Detection of the Transcriptional Regulation of OCT4 by Selected Genes

The all-in-one CRISPRa plasmid of the dCas9-SAM system, containing the dCas9-VP64, MS2-p65-HSF1, and sgRNA components, was provided by the laboratory of cell and genetic engineering, Northeast Agricultural University. We cloned the sgRNA of SOX2, KLF4, and PRDM14 (Supplementary Table S2-2) into the downstream region of the U6 promoter to construct CRISPR-active plasmids. The dCas9-SAM system was transfected into the PK15 cell line by Lipofectamine 3000 (ThermoFisher, Shanghai, China). After 48 h, G418 (Sigma, Shanghai, China) was added for cell screening. The positive cell colonies were picked up for the detection of RNA expression of the targeted genes and the OCT4 gene.

### 2.9. Real-Time Quantitative PCR

For 1 × 10^6^ cells, we took 5 μg plasmid DNA and supplemented it with opti-MEM (Gibco, Grand Island, NY, USA) to a volume of 20 μL. After the mixed liquid was prepared, we used the program set by the electric rotation instrument for electric rotation. After electroporation, the cells were connected to the culture dish for culturing. After drug screening, the cells were collected for RNA extraction.

Total RNA was extracted using Trizol (Takara, Dalian, China). Briefly, we added 1 mL of Trizol reagent to every 5 × 10^6^ cells and homogenized the sample. This was then incubated at room temperature for 5 min before adding 400 μL chloroform to each 1 mL of Trizol reagent and mixing the whole sample upside down and incubating it in ice for 20 min. Then, we centrifuged the sample at 10,000× *g* for 15 min at 4 °C and transferred the aqueous phase containing RNA to a new EP tube. We then added 500 μL of isopropanol to the aqueous phase for each 1 mL of Trizol reagent used for lysis and incubated in ice for 10 min. This was centrifuged at 10,000× *g* for 10 min at 4 °C, and we then discarded the supernatant. Next, we resuspended the precipitate in 1 mL of 75% ethanol. We then vortexed the sample, centrifuged it at 7500× *g* for 5 min at 4 °C, discarded the supernatant, and dried the RNA for 10 min. Finally, we resuspended the pellet with 30 μL RNase-free water with repeated blows and incubated the whole mixture at 55 °C for 10 min. The cDNA was generated via reverse transcription using a high-capacity cDNA reverse transcription kit (Applied Biosystems, Waltham, MA, USA). Using TB green™, Q-PCR was performed with premixed EX Taq (Takara, Dalian, China) and a 7500 Q-PCR system (Applied Biosystems, USA). The CT value was calculated after the reaction was completed, and the amount of target sequence normalized to the reference sequence was calculated as 2^−ΔΔCT^. The data are expressed as the mean ± standard deviation (SD). See Supplementary Table S2-3 for Q-PCR primers.

### 2.10. Statistical Analysis

SPSS 16.0 Statistical Software was used to conduct the statistical analysis (SPSS, Inc., Chicago, IL, USA). All results are represented as the mean ± SE of at least three distinct repeated experiments. The *t*-test was used to compare differences. The statistical significance of the difference was defined as a value of *p* < 0.05.

## 3. Results

### 3.1. Design and Construction of Porcine Transcription Factor sgRNA Library

**Figure 1:**
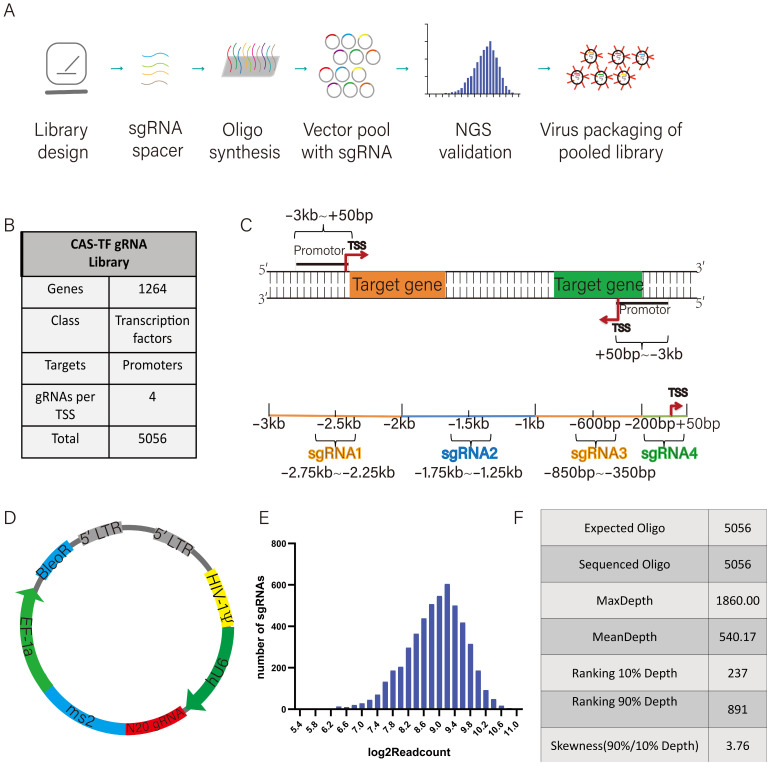
Construction of porcine transcription factor CRISPR-activated sgRNA library. (**A**) Schematic of construction of CRISRP-activated sgRNA library. (**B**) The composition of the CRISRP-activated sgRNA library. (**C**) The location information of the sgRNAs in the gene promoter region. (**D**) The schematic of the pLenti-sgRNA (MS2) vector. (**E**) The distribution of the sgRNA read count in the CRISPR-activated sgRNA library. (**F**) The NGS sequencing results of the CRISPR-activated sgRNA library.

SgRNA library construction typically includes library design, oligonucleotide synthesis, library vector construction, plasmid amplification, NGS validation, and virus packaging (Figure 1A). To obtain information regarding all transcription factors in the pig genome, we referred to the information of human transcription factors and compared this with the pig genome to select a total of 1264 transcription factors for the construction of this transcriptional activation library (Figure 1B). Four sgRNAs were designed for each of the transcription factors from 3000 bp upstream to 50 bp downstream in each transcription start region (Figure 1C). A total of 5056 sgRNAs were used to construct a targeted gRNA library (Supplementary Table S1). The sgRNA fused with an MS2 stem loop was cloned into the lentiviral vector pLenti_sgRNA (MS2) (Figure 1D). The recombinant sgRNA library was sequenced to identify its diversity. The NGS results show that the distribution of the sgRNA libraries is relatively uniform, with a maximum depth of 1860 and an average depth of 540.17, along with a coverage rate of 100% (Figure 1E,F). The synthesized sgRNA vector was packaged as a lentiviral library with a titer of 2.51 × 10^8^ IFU/mL and prepared for subsequent cell infection experiments.

### 3.2. Establishment of Porcine Cell Lines of a Porcine OCT4 Promoter-Driven EGFP Reporter and dCas9-SAM for CRISPRa Screening

**Figure 2:**
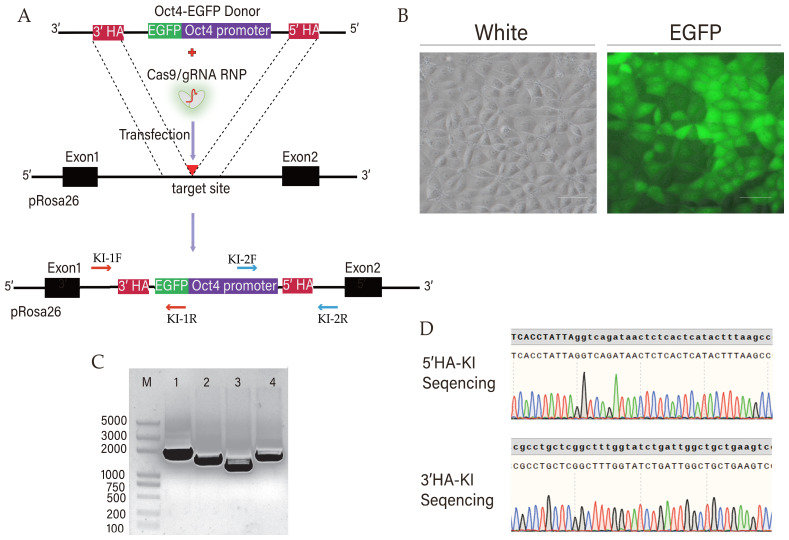
Construction of cell lines expressing porcine OCT4 promoter-driven EGFP. (**A**) Schematic diagram of pOCT4-EGFP knock-in at porcine ROSA26 and PCR amplification site. (**B**) The expression of EGFP under pOCT4 promoter at porcine ROSA26 site. (**C**) Electrophoresis of PCR products. LaneM is the DNA marker. The length of Lane1 is 1778 bp, and the length of Lane2 is 1474 bp. Lane3 and -4 are 3-end PCR products: Lane3 is 1207 bp, and Lane4 is 1591 bp. (**D**) Sequencing results of pOCT4-EGFP knock-in DNA fragment.

In order to screen transcription factors that regulate OCT4, we connected the 3000 bp region of the pig OCT4 promoter to the EGFP protein to construct a reporter system for pig OCT4 promoter-driven EGFP expression. To ensure the copy number of the pOCT4-EGFP sequence, we used CRISPR/Cas9 technology to integrate the sequence into the pig ROSA26 site (Figure 2A). The expression of the EGFP fluorescent protein could be detected under a fluorescence microscope, indicating that the inserted pig pOCT4 promoter can function normally (Figure 2B). PCR amplification and DNA sequencing were used to confirm the insertion of the ROSA26 site sequence, indicating that the pOCT4 EGFP sequence was correctly inserted into the pig ROSA26 site (Figure 2C,D).

**Figure 3:**
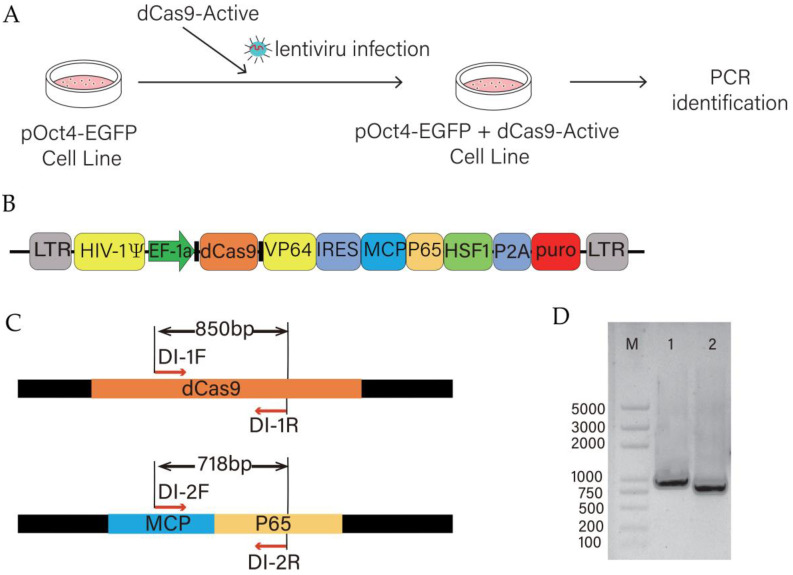
Construction of PK15 cell lines expressing dCas9 and activating elements. (**A**) Schematic diagram of dCas9-SAM system elements infecting the cell line. (**B**) Plasmid pattern diagram expressing components of the dCas9-SAM system. (**C**) Schematic diagram of the PCR amplification region. (**D**) Electrophoresis of lentivirus integration results identified by PCR. LaneM is the DNA marker. Lane1 is an 850 bp amplification product and Lane2 is a 718 bp amplification product.

The dCas9-SAM system expressing dCas9-VP64 and MCP-P65-HSF1 elements was packaged as a lentivirus (Figure 3A,B). After infecting pOCT-EGFP cell lines with dCas9-SAM lentivirus, we confirmed that dCas9-SAM was stably integrated into the cell genome through PCR amplification and DNA sequencing (Figure 3C,D). In this way, we obtained a cell line that expresses both pOCT4-EGFP and dCas9-SAM systems simultaneously.

### 3.3. CRISPRa Screening for the Transcription Factors Regulating the Porcine OCT4 Gene

**Figure 4:**
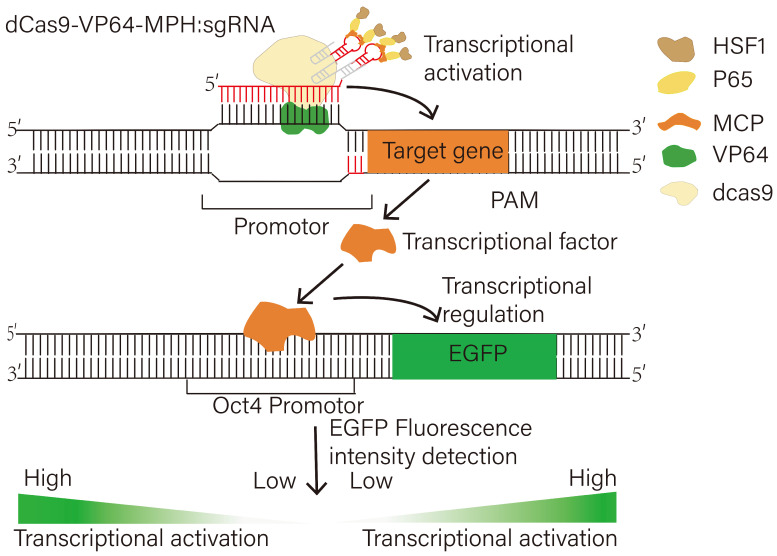
Schematic of screening transcription factors regulating OCT4 gene based on CRISPR activation technology.

In this study, the Synergistic Activation Mediator (SAM) library, which contained a dCas9-VP64 fusion protein combined with a modified sgRNA and a trimeric fusion protein complex, was used to increase the expression of TFs. The modified sgRNA sequence contained two RNA hairpin aptamers recognized by the bacteriophage coat protein MS2. The trimeric fusion protein was made up of the MS2 protein for binding the sgRNA and two transcription activation domains from NF-jB (p65) and heat shock factor 1 (HSF1). Then, sgRNA binding with dCas9 was used to specifically identify and target the promoter of the target gene. The combination of the VP64, p65, and HSF1 activation domains synergistically increased the gene expression of the targeted TF. After the target TFs were translated into a transcription factor protein, they could bind to the OCT4 promoter region and upregulate or downregulate the expression of OCT4 (Figure 4). To detect the regulatory effects of transcription factors on OCT4, we constructed a porcine OCT4 promoter driving EGFP expression in the knock-in cell line, so the role of transcription factors can be identified according to changes in the fluorescence intensity of EGFP. If the intensity of green fluorescence was found to have increased, this would indicate that the transcription factors had an active effect on OCT4 expression. Conversely, if the intensity of green fluorescence decreased, this suggested that the activated transcription factors exerted an inhibitory effect on OCT4 expression (Figure 4).

**Figure 5:**
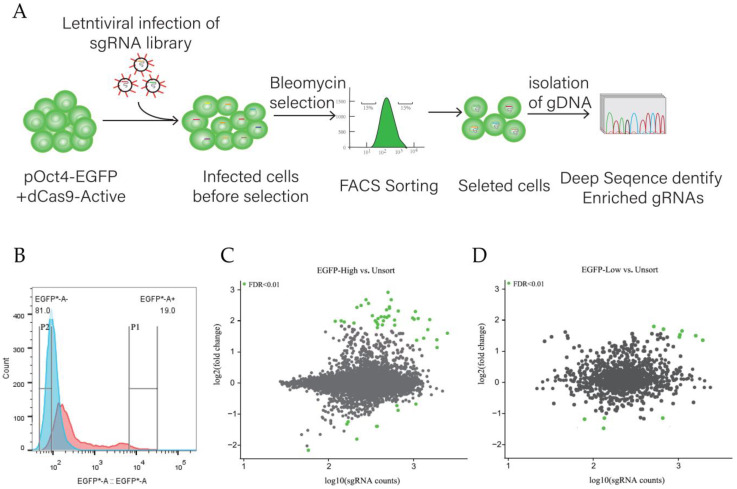
CRISPRa library was used to screen transcription factors regulating the OCT4 gene. (**A**) Schematic diagram of CRISPRa library screening process. (**B**) The results of flow cytometry screening for library infection showing that the cell population corresponding to P1 was the 15% with the highest fluorescence intensity, and that corresponding to P2 was the 15% with the lowest fluorescence intensity. * *p* < 0.05. (**C**) Analysis of sgRNA enrichment results of high-fluorescence intensity cell population. (**D**) Analysis of sgRNA enrichment results of low-fluorescence intensity cell population.

To identify a set of OCT4 regulators in an unbiased manner, we performed CRISPRa pooled gRNA screening in the pOCT4-EGFP and dCas9-SAM cell lines (Figure 5A). The CRISPRa gRNA lentiviral library was transduced at a multiplicity of infection (MOI) of 0.2 and at 550-fold coverage of the library to ensure that most cells activated a single TF. After 3 days of bleomycin selection and gRNA expression, we used FACS to isolate the top and bottom 15% of EGFP-expressing cells (Figure 5A,B) and quantified gRNA abundance with differential expression analysis following the deep sequencing of the protospacers within each sorted bin. The transcription factors that regulated the expression of the OCT4 gene were identified by analyzing the enrichment of sgRNA (Figure 5A). The results show that, compared with the control group, the cells infected with the sgRNA library had a cell peak with higher fluorescence intensity (P1) and a cell peak with lower fluorescence intensity (P2) after flow cytometry screening, indicating that transcription factors activated by the sgRNA library could activate or inhibit OCT4 (Figure 5B). Through the analysis of the amplified sgRNA sequence sequencing results, we found 31 transcription factors enriched in the 15% of cells with the highest fluorescence intensity, among which KLF4, SOX2, PRDM14, KLF2, and GATA4 were the most enriched (Figure 5C, Supplementary Table S3). In the 15% of cells with the lowest fluorescence intensity, five transcription factors were found enriched, including NR2F2, NR2F1, TCF3, OTX2, and CDX2 (Figure 5D).

### 3.4. Validation of Candidate Transcription Factors of OCT4 by CRISPR Active System

**Figure 6:**
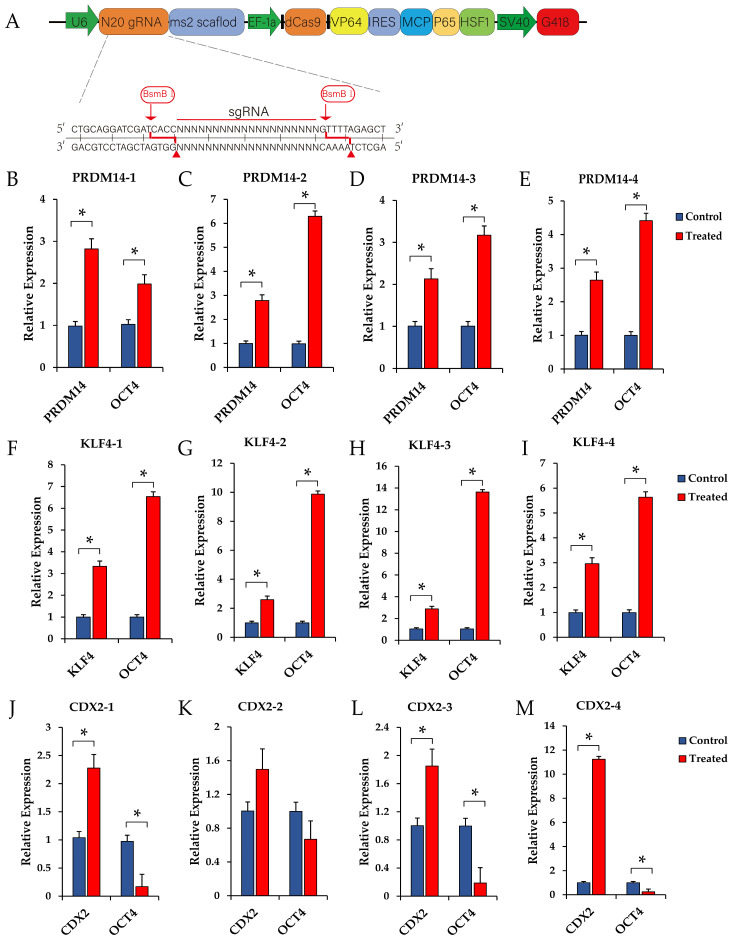
Verification of the transcriptional regulation of the OCT4 gene by CRISPR-activated transcription factors. (**A**) Schematic diagram of all-in-one CRISPR activation vector. (**B**–**E**) Verification of the transcriptional regulation of the OCT4 gene by CRISPR-activated PRDM14 gene with different sgRNAs. (**F**–**I**) Verification of the transcriptional regulation of the OCT4 gene by CRISPR-activated KLF4 gene with different sgRNAs. (**J**–**M**) Verification of the transcriptional regulation of the OCT4 gene by CRISPR-activated CDX2 gene with different sgRNAs.* *p* < 0.05.

To validate the activities of the candidate OCT4 regulator TFs, we individually tested the most enriched gRNA for the three TFs, including PRDM14, KLF4, and CDX2, identified in the CAS-TF screen. All four sgRNAs in the CAS-TF library of these genes were cloned into the “all-in-one” activation vector, separately (Figure 6A). The expressions of the activation-targeted gene and OCT4 gene were detected, after which the “all-in-one” activation vector was transferred into pig PK15 cells. The results show that sgRNAs targeting the PRDM14 and KLF4 genes effectively activated the expression of the PRDM14 and KLF4 genes, and compared with the control group, the expression of the OCT4 gene was also found to be increased (Figure 6B–I). These results indicate that elevated expression levels of PRDM14 and KLF4 can both upregulate the expression of the OCT4 gene. Three sgRNAs targeting the CDX2 gene significantly activated the expression of CDX2, while the corresponding expression of OCT4 was also significantly reduced (Figure 6J–M). These results indicate that CDX2 has an inhibitory effect on the expression of the OCT4 gene. The above results are consistent with the results of library screening.

## 4. Discussion

In the post-genomic era, following the completion of the sequencing of the porcine genome, there is a need for methods that can stabilize and broadly interfere with gene expression for gene function exploration [5]. Here, we established a pig transcription factor CRISPR-activated sgRNA library, in which 5056 sgRNAs were designed, targeting 1246 transcription factors and covering a set of putative transcription factors in pigs. Although several genome-wide knockout libraries have been established in pigs, such as RNA interference-based and RNA-directed CRISPR-Cas9 knockout libraries, the full transcription factor CRISPR-activated library that we constructed is the first to be reported [34,35,36].

The CRISPR-activated sgRNA library can be used to rapidly screen for gain-of-function phenotypes in a pooled format [12]. It was reported that human or mouse CRISPR library screens have been successfully used to identify many previously unknown phenotypes or complex biological processes [15,16,17,37]. Previously, gain-of-function screens were primarily limited to cDNA overexpression libraries, which suffered from incomplete representation, overexpression beyond physiological levels and endogenous regulation, lack of isoform diversity, and high costs of construction [12,38]. CRISPRa overcomes these limitations because the CRISPRa sgRNA library is a high-throughput tool used to induce and activate multiple genes at the endogenous locus, and it simply requires the synthesis and cloning of RNA guides, making it much more affordable [39].

CRISPR screening technology is a genome-wide CRISPR technology based on the CRISPR-Cas9 system combined with NGS technology [7,8]. The experimental process of the CRISPR screening technology consists of multiple steps; first of all, sgRNA library sequences need to be designed [6]. In this study, the pig transcription factor CRISPR-activated library contains 1246 transcription factors, covering all transcription factors in pigs. To ensure effective gene activation, sgRNA was designed from 3000 bp upstream to 50 bp downstream of the TSS. Previous studies have shown that the low activation efficiency of a single sgRNA can be overcome by binding dCas9-VP64 to sgRNAs in the proximal promoter region of the target gene, which is located in a region about 1000 bp upstream of the TSS [6,7,8,10]. Therefore, the upstream 3000 bp region selected is sufficient to include the promoter region. As the region extending from 200 bp upstream to 50 bp downstream of the TSS is the core promoter region, the distance between sgRNA target sites and the TSS can reach the highest activation level within the range of 200 bp downstream to 1 bp upstream [6]. Therefore, when designing the four sgRNA subregions of each transcription factor, one sgRNA was designed in the 200 bp upstream to 50 bp downstream region. Meanwhile, to ensure the four sgRNAs were evenly distributed within 3000 bp, the remaining three sgRNAs were designed to be located in different regions for every 1000 bp, such that the transcriptional activation complex could be fully bound to different regions of the promoter, and the level of activated genes could be upregulated [7,8,38]. The creation of this comprehensive pig transcription factor CRISPR library enables high-throughput studies on gene regulation for both basic and applied research in pigs. This library can facilitate further exploration into the mechanisms governing gene expression, with potential implications for improving pig breeding and advancing agricultural biotechnology.

In addition to designing sgRNAs in the gene promoter region for transcriptional activation, all possible sgRNA targets with the Cas9 ortholog-specific protospacer-adjacent motif (PAM) were identified and selected based on the GC content, MIT value, and base polymerization of the sgRNA. The GC content of each sgRNA was between 30% and 70%, ensuring the effective binding of the sgRNA [6,38]. The MIT value was greater than 60, and the highest score was selected when other screening criteria were met, which ensured the minimum miss efficiency, and there were no sets of four consecutive As and Ts, thus effectively precluding the termination of transcription. Through four screening principles, sgRNA with a high activation effect could be selected to activate target genes [10,40]. Although the specificity and efficiency of sgRNAs can be guaranteed through design, false-positive sgRNAs may still arise during screening. In this study, by designing four sgRNAs for each gene in the library, the presence of false-positive sgRNAs was reduced. Further, when validating the selected targeted sgRNAs, it was required that multiple different sgRNAs targeting the same gene display the same phenotype. The sgRNA libraries were synthesized after being designed, and lentiviral plasmid libraries were constructed. Throughout the sgRNA library cloning and amplification process, it is important to minimize any potential bias that may affect the enrichment of the sgRNA. The number of PCR cycles in the initial amplification of the pooled oligo library synthesis should be limited to prevent introducing bias during amplification [18]. We scaled each step of the cloning procedure provided according to the size of the library to reduce the loss of sgRNA representation [18]. In this study, the coverage and homogeneity of the plasmid libraries were quality-controlled by NGS sequencing.

Plasmid libraries are packaged with viruses and then transfected with cells; at this time, it is necessary to ensure that a cell is infected by only one lentivirus, so as to prevent the editing of multiple genes in a single cell from affecting the subsequent analysis of the data and the interpretation of the results. Therefore, it is necessary to evaluate and select the most appropriate MOI for the experiment, and a relatively low MOI (0.3–0.5) is usually used to ensure that a cell mainly receives only one virus particle [6,10,41]. After PCR amplification, the number of reads enriched for each sgRNA was counted using high-throughput sequencing technology. By comparing the sgRNA sequence given with the designed sgRNA in the screened library, we obtained candidate target genes corresponding to activation [6,10,41].

When constructing PK15 cell lines for the screening of transcription factors, this study used CRISPR technology to successfully perform site-specific insertion at the ROSA26 site in pigs, resulting in the pig OCT4 promoter driving EGFP gene expression, thus knocking into PK15 cells. The ROSA26 site is known to express the exogenous gene without adverse effects and is highly conserved with relatively clear location information in pigs [42,43]. Studies have proven that gene knock-in at this site in pigs can effectively lead to the expression of genes and produce transgenic pigs [42,43]. In this study, sgRNA was designed at the first intron of ROSA26 for targeted knock-in. To prevent the promoter of ROSA26 from driving the expression of EGFP and avoid interference with the screening results, the knock-in direction of the OCT4 promoter was set as opposite to the transcription direction of ROSA26.

OCT4 is a transcription factor that is highly expressed in early embryos and plays a key role in the maintenance and self-renewal of pluripotency in different species [4,23,24,44]. By sorting the 15% of cells with the strongest fluorescence intensity, sgRNAs corresponding to KLF4, SOX2, PRDM14, KLF2, and GATA4 transcription factors were found to be significantly richer. These results indicate that the transcription factors KLF4, SOX2, PRDM14, KLF2, and GATA4 could activate OCT4 expression. It has been documented that SOX2, KLF4, and PRDM14 are indeed regulatory factors of OCT4 activation [21,44,45]. Similarly, by sorting the 15% of cells with the weakest fluorescence intensity, sgRNAs corresponding to the NR2F2, NR2F1, TCF3, OTX2, and CDX2 transcription factors were found to be significantly enriched. This indicates that the NR2F2, NR2F1, TCF3, OTX2, and CDX2 transcription factors can inhibit the expression of OCT4 [21,44,45]. Cdx2 was found to inhibit OCT4 expression when overexpressed in mouse embryos [46]. NR2F1 binds to OCT4-specific retinoic acid response elements and inhibits OCT4 expression [47]. Meanwhile, the activation effects of KLF4 and PRDM14 on OCT4 and the inhibition effects of CDX2 on OCT4 were also confirmed by fluorescence quantitative PCR technology, further proving the effectiveness of sgRNA library screening. It is worth noting that GATA4, as a strong transcription factor that binds to the GATA motif in the upstream regulatory region of OCT4, activates OCT4 expression in TE in pigs [48]. In mice, no evidence exists that GATA family proteins have a direct regulatory relationship with OCT4 [43]. These results indicate that the regulation of OCT4 by the transcription factor GATA4 is species-specific, which is worthy of further study. In this study, PK15 cells were used, while OCT4 is generally expressed in pluripotent stem cells [49]. Therefore, the genes screened in this study cannot be used to represent all the genes that regulate OCT4. In future studies, we will screen for transcription factors that regulate OCT4 in pig pluripotent stem cells.

## 5. Conclusions

In summary, this study has established a library of CRISPR-activated pig transcription factors and widely screened the transcriptional regulators that regulate OCT4 by constructing sgRNA libraries. The CRISPRa sgRNA library is a powerful tool that can be used to understand the regulatory network of a desired cell type, as well as for discovering factors that can promote desired traits in pigs.

## Data Availability

The authors declare that all data supporting the findings of this study are available within the paper and its Supplementary Materials.

## Supplementary Materials

The following supporting information can be downloaded at: https://www.mdpi.com/article/10.3390/ani15010019/s1. Table S1. List of 1264 transcription factors; Table S2. The primers used in this paper; Table S3. Transcription factors with activation effect.

## References

[1] Shi L.J., Li H.H., Wang L.X. (2023). Genetic parameters estimation and genome molecular marker identification for gestation length in pigs. Front. Genet..

[2] Rovito D., Rerra A.I., Ueberschlag-Pitiot V., Joshi S., Karasu N., Dacleu-Siewe V., Ben Rayana K., Ghaibour K., Parisotto M., Ferry A. (2021). Myod1 and GR coordinate myofiber-specific transcriptional enhancers. Nucleic Acids Res..

[3] Siersbæk R., Nielsen R., Mandrup S. (2010). PPARγ in adipocyte differentiation and metabolism—Novel insights from genome-wide studies. FEBS Lett..

[4] Fogarty N.M.E., McCarthy A., Snijders K.E., Powell B.E., Kubikova N., Lakeley P.B., Lea R., Lder K.E., Wamaitha S.E., Kim D. (2017). Genome editing reveals a role for OCT4 in human embryogenesis. Nature.

[5] Durosaro S.O., Iyasere O.S., Ilori B.M., Oyeniran V.J., Ozoje M.O. (2023). Molecular regulation, breed differences and genes involved in stress control in farm animals. Domest. Anim. Endocrinol..

[6] Konermann S., Brigham M.D., Trevino A.E., Joung J., Abudayyeh O.O., Barcena C., Hsu P.D., Habib N., Gootenberg J.S., Nishimasu H. (2015). Genome-scale transcriptional activation by an engineered CRISPR-Cas9 complex. Nature.

[7] Maeder M.L., Linder S.J., Cascio V.M., Fu Y., Ho Q.H., Joung J.K. (2013). CRISPR RNA-guided activation of endogenous human genes. Nat. Methods.

[8] Perez-Pinera P., Kocak D.D., Vockley C.M., Adler A.F., Kabadi A.M., Polstein L.R., Thakore P.I., Glass K.A., Ousterout D.G., Leong K.W. (2013). RNA-guided gene activation by CRISPR-Cas9-based transcription factors. Nat. Methods.

[9] Gilbert L.A., Horlbeck M.A., Adamson B., Villalta J.E., Chen Y., Whitehead E.H., Guimaraes C., Panning B., Ploegh H.L., Bassik M.C. (2014). Genome-Scale CRISPR-Mediated Control of Gene Repression and Activation. Cell.

[10] Chavez A., Scheiman J., Vora S., Pruitt B.W., Tuttle M., Iyer E.P.R., Lin S.L., Kiani S., Guzman C.D., Wiegand D.J. (2015). Highly efficient Cas9-mediated transcriptional programming. Nat. Methods.

[11] Wang T., Wei J.J., Sabatini D.M., Lander E.S. (2014). Genetic Screens in Human Cells Using the CRISPR-Cas9 System. Science.

[12] Didovyk A., Borek B., Tsimring L., Hasty J. (2016). Transcriptional regulation with CRISPR-Cas9: Principles, advances, and applications. Curr. Opin. Biotechnol..

[13] Liu Y., Yu C., Daley T.P., Wang F., Cao W.S., Bhate S., Lin X., Still C., Liu H., Zhao D. (2018). CRISPR Activation Screens Systematically Identify Factors that Drive Neuronal Fate and Reprogramming. Cell Stem Cell.

[14] Makda G., Jason N., Benjamin G. (2018). CRISPR–Cas9 Genetic Analysis of Virus–Host Interactions. Viruses.

[15] Yang J., Rajan S.S., Friedrich M.J., Lan G., Zou X., Ponstingl H., Garyfallos D.A., Liu P., Bradley A., Metzakopian E. (2019). Genome-Scale CRISPRa Screen Identifies Novel Factors for Cellular Reprogramming. Stem Cell Rep..

[16] Black J.B., McCutcheon S.R., Dube S., Barrera A., Klann T.S., Rice G.A., Adkar S.S., Soderling S.H., Reddy T.E., Gersbach C.A. (2020). Master Regulators and Cofactors of Human Neuronal Cell Fate Specification Identified by CRISPR Gene Activation Screens. Cell Rep..

[17] Alda-Catalinas C., Bredikhin D., Hernando-Herraez I., Santos F., Reik W. (2020). A Single-Cell Transcriptomics CRISPR-Activation Screen Identifies Epigenetic Regulators of the Zygotic Genome Activation Program. Cell Syst..

[18] Joung J., Konermann S., Gootenberg J.S., Abudayyeh O.O., Platt R.J., Brigham M.D., Sanjana N.E., Zhang F. (2017). Genome-scale CRISPR-Cas9 knockout and transcriptional activation screening. Nat. Protoc..

[19] Cai R., Lv R., Shi X.e., Yang G., Jin J. (2023). CRISPR/dCas9 Tools: Epigenetic Mechanism and Application in Gene Transcriptional Regulation. Int. J. Mol. Sci..

[20] Varzideh F., Gambardella J., Kansakar U., Jankauskas S.S., Santulli G. (2023). Molecular Mechanisms Underlying Pluripotency and Self-Renewal of Embryonic Stem Cells. Int. J. Mol. Sci..

[21] Li Y.Q. (2017). Networks of Transcription Factors for Oct4 Expression in Mice. DNA Cell Biol..

[22] Rizzino A., Wuebben E.L. (2016). Sox2/Oct4: A delicately balanced partnership in pluripotent stem cells and embryogenesis. Biochim. Et Biophys. Acta Gene Regul. Mech..

[23] Simmet K., Kurome M., Zakhartchenko V., Reichenbach H.D., Springer C., Bähr A., Blum H., Philippou-Massier J., Wolf E. (2022). OCT4/POU5F1 is indispensable for the lineage differentiation of the inner cell mass in bovine embryos. FASEB J..

[24] Frum T., Halbisen M.A., Wang C.Y., Amiri H., Robson P., Ralston A. (2013). Oct4 Cell-Autonomously Promotes Primitive Endoderm Development in the Mouse Blastocyst. Dev. Cell.

[25] Bakhmet E.I., Tomilin A.N. (2021). Key features of the POU transcription factor Oct4 from an evolutionary perspective. Cell. Mol. Life Sci..

[26] Matys V., Fricke E., Geffers R., Gössling E., Haubrock M., Hehl R., Hornischer K., Karas D., Kel A.E., Kel-Margoulis O.V. (2003). TRANSFAC^®^: Transcriptional regulation, from patterns to profiles. Nucleic Acids Res..

[27] Matys V., Kel-Margoulis O.V., Fricke E., Liebich I., Land S., Barre-Dirrie A., Reuter I., Chekmenev D., Krull M., Hornischer K. (2006). TRANSFAC^®^ and its module TRANSCompel^®^: Transcriptional gene regulation in eukaryotes. Nucleic Acids Res..

[28] Zhao Y.B. (2023). TFSyntax: A database of transcription factors binding syntax in mammalian genomes. Nucleic Acids Res..

[29] Warr A., Affara N., Aken B., Beiki H., Bickhart D.M., Billis K., Chow W., Eory L., Finlayson H.A., Flicek P. (2020). An improved pig reference genome sequence to enable pig genetics and genomics research. GigaScience.

[30] Huang L.Z., Fan N.N., Cai J., Yang D.S., Zhao B.T., Ouyang Z., Gu W.W., Lai L.X. (2011). Establishment of a Porcine Oct-4 Promoter-Driven EGFP Reporter System for Monitoring Pluripotency of Porcine Stem Cells. Cell. Reprogramming.

[31] Xie Z., Pang D., Wang K., Li M., Guo N., Yuan H., Li J., Zou X., Jiao H., Ouyang H. (2017). Optimization of a CRISPR/Cas9-mediated Knock-in Strategy at the Porcine Rosa26 Locus in Porcine Foetal Fibroblasts. Sci. Rep..

[32] Li W., Xu H., Xiao T.F., Cong L., Love M.I., Zhang F., Irizarry R.A., Liu J.S., Brown M., Liu X.S. (2014). MAGeCK enables robust identification of essential genes from genome-scale CRISPR/Cas9 knockout screens. Genome Biol..

[33] Love M.I., Huber W., Anders S. (2014). Moderated estimation of fold change and dispersion for RNA-seq data with DESeq2. Genome Biol..

[34] Yu C., Zhong H., Yang X., Li G., Wu Z., Yang H. (2021). Establishment of a pig CRISPR/Cas9 knockout library for functional gene screening in pig cells. Biotechnol. J..

[35] Dang W., Li T., Xu F., Wang Y., Yang F., Zheng H. (2022). Establishment of a CRISPR/Cas9 knockout library for screening type I interferon-inducible antiviral effectors in pig cells. Front. Immunol..

[36] Zhao C., Liu H., Xiao T., Wang Z., Zhao S. (2020). CRISPR screening of porcine sgRNA library identifies host factors associated with Japanese encephalitis virus replication. Nat. Commun..

[37] Saratov V., Ngo Q.A., Pedot G., Sidorov S., Wachtel M., Niggli F.K., Schäfer B.W. (2022). CRISPR activation screen identifies TGFβ-associated PEG10 as a crucial tumor suppressor in Ewing sarcoma. Sci. Rep..

[38] Rusk N. (2015). Genomics: CRISPR gain-of-function screens. Nat. Methods.

[39] Kampmann M. (2018). CRISPRi and CRISPRa Screens in Mammalian Cells for Precision Biology and Medicine. ACS Chem. Biol..

[40] Miles L.A., Garippa R.J., Poirier J.T. (2016). Design, execution, and analysis of pooled in vitro CRISPR/Cas9 screens. FEBS J..

[41] Shalem O., Sanjana N.E., Hartenian E., Shi X., Scott D.A., Mikkelsen T.S., Heckl D., Ebert B.L., Root D.E., Doench J.G. (2014). Genome-Scale CRISPR-Cas9 Knockout Screening in Human Cells. Science.

[42] Kong Q.R., Hai T., Ma J., Huang T.Q., Jiang D.D., Xie B.T., Wu M.L., Wang J.Q., Song Y.R., Wang Y. (2014). ROSA26 Locus Supports Tissue-Specific Promoter Driving Transgene Expression Specifically in Pig. PLoS ONE.

[43] Li X.P., Yang Y., Bu L., Guo X.G., Tang C.C., Song J., Fan N.N., Zhao B.T., Ouyang Z., Liu Z.M. (2014). ROSA26-targeted swine models for stable gene over-expression and Cre-mediated lineage tracing. Cell Res..

[44] Sánchez-Sánchez A.V., Camp E., García-España A., Leal-Tassias A., Mullor J.L. (2010). Medaka Oct4 Is Expressed During Early Embryo Development, and in Primordial Germ Cells and Adult Gonads. Dev. Dyn..

[45] Boiani M., Scholer H.R. (2005). Regulatory networks in embryo-derived pluripotent stem cells. Nat. Rev. Mol. Cell Biol..

[46] Niwa H., Toyooka Y., Shimosato D., Strumpf D., Takahashi K., Yagi R., Rossant J. (2005). Interaction between Oct3/4 and Cdx2 Determines Trophectoderm Differentiation. Cell.

[47] Wang J.L., Park J.W., Drissi H., Wang X.F., Xu R.H. (2014). Epigenetic Regulation of miR-302 by JMJD1C Inhibits Neural Differentiation of Human Embryonic Stem Cells. J. Biol. Chem..

[48] Bou G., Guo J., Liu S., Guo S., Davaakhuu G., Lv Q., Xue B., Qiao S., Lv J., Weng X. (2022). OCT4 expression transactivated by GATA protein is essential for non-rodent trophectoderm early development. Cell Rep..

[49] Tolen E.A., Xiong L., Choi J., Velychko S., Caizzi L., Velychko T., Adachi K., MacCarthy C.M., Lidschreiber M., Cramer P. (2022). Oct4 differentially regulates chromatin opening and enhancer transcription in pluripotent stem cells. eLife.

