# Supplementary material for: Establishment of a CRISPR-Based Lentiviral Activation Library for Transcription Factor Screening in Porcine Cells
Source: Animals (Basel). 2024 Dec 25;15(1):19. doi: 10.3390/ani15010019 (PMC11718943; doi:10.3390/ani15010019)
Supplement: Supplementary file 1 [file animals-15-00019-s001.zip › Supplementary Table S2. QPCR primers and gene knock-in identification primers.pdf]

Supplementary Table S2-1. primers of gene knock-in identification

Primer ID	Sequence(5’-3’)
**3‘KI-1778bp-F**	GAAGTCGGGAGCATATCGTTTGTTACGCTGGAAG
**3’KI-1778bp-R**	GAGCGAACGACCTACACCGAACTGAGATACCTAC
**3’KI-1474bp-F**	GCTTCCTGCTCTTCTCTTGTCACTGATTGG
**3’KI-1474bp-R**	GCCTGGTATCTTTATAGTCCTGTCGGGTTTC
**5’KI-1207bp-F**	GCCCTACACAAAGACAAATGCCAAGTATCTG
**5’KI-1207bp-R**	ACAACCTGATAAGTGCTATGATTCAACCTGCTATG
**5’KI-1591bp-F**	GTTAAGTCATCAGACCTGGCTTCTCCACTCT
**5’KI-1591bp-R**	AGGACTATACTAGGTGCTTAGGTACAAGACTCAACA
**SAM-850bp-F**	ATTATCAAGGACAAGGACTTCCTGGACAAT
**SAM-850bp-R**	AGTAGTTCTTCATCTTCTTCACGACCTCTTC
**SAM-718bp-F**	TTCTGACTGTGAACTCATCGTGAAGGCAAT
**SAM-718bp-R**	CTGTGACAGCAGGGCACTAAAGTCCATATC

Supplementary Table S2-2. sgRNAs of KLF4, CDX2 and PRDM14

SgRNA ID	Sequence(5’-3’)
**KLF4.1**	CCAACTAATGCAGTTTGTGC
**KLF4.2**	CTCGCCCCGACGAATCCATT
**KLF4.3**	CGTACCTCTCTTACGCACGC
**KLF4.4**	TATAAGTAAGGAGCGCGCGG
**CDX2.1**	CAGTGAAGCCTATGCTCGCT
**CDX2.2**	TCGCGGTCGCTCAACTGAGA
**CDX2.3**	CAACGTTTCTGGCCAACCCG
**CDX2.4**	GCATGGTGAGGTTTGCCGTC
**PRDM14.1**	CAAATGTCCTAAGTCGGCAA
**PRDM14.2**	AGTCCCGGCCTTATGCGCGT
**PRDM14.3**	CGTTATCCTACCCGAGCTCG
**PRDM14.4**	ATCCAATTCCCTTCAACCCG

Supplementary Table S2-3. primers of RT-PCR

Primer ID	Sequence(5’-3’)
**qCDX2-F**	TCCGCATCCACTCGCACA
**qCDX2-R**	GAGGACTGGAACGGCTACGC
**qKLF4-F**	CATGAGTTGGGGGAGGGAAG
**qKLF4-R**	ACTCACCAAGCACCATCGTT
**qPRDM14-F**	AGGGTACAGATGTGAAAGATGTGGAA
**qPRDM14-R**	TGCTTGTTTAGGCTGGAAGATTGAG
**qOCT4-F**	GTGTTCAGCCAAACGACCATC
**qOCT4-R**	GTCTCTGCCTTGCATATCTCC
**qGAPDH-F**	CTCAACGGGAAGCTCACTGG
**qGAPDH-R**	TGATGTCATCATATTTGCAGGTT
